# ^1^H, ^13^C, and ^15^N resonance assignments of a conserved putative cell wall binding domain from *Enterococcus faecalis*

**DOI:** 10.1007/s12104-022-10087-2

**Published:** 2022-06-04

**Authors:** Jessica L. Davis, Andrea M. Hounslow, Nicola J. Baxter, Stéphane Mesnage, Mike P. Williamson

**Affiliations:** School of Biosciences, University of Sheffield, Firth Court, Western Bank, S10 2TN Sheffield, UK

**Keywords:** Peptidoglycan, Hydrolase, *E. faecalis*, AtlE, R6

## Abstract

*Enterococcus faecalis* is a major causative agent of hospital acquired infections. The ability of *E. faecalis* to evade the host immune system is essential during pathogenesis, which has been shown to be dependent on the complete separation of daughter cells by peptidoglycan hydrolases. AtlE is a peptidoglycan hydrolase which is predicted to bind to the cell wall of *E. faecalis*, via six C-terminal repeat sequences. Here, we report the near complete assignment of one of these six repeats, as well as the predicted backbone structure and dynamics. This data will provide a platform for future NMR studies to explore the ligand recognition motif of AtlE and help to uncover its potential role in *E. faecalis* virulence.

## Biological context

**Fig. 1:**
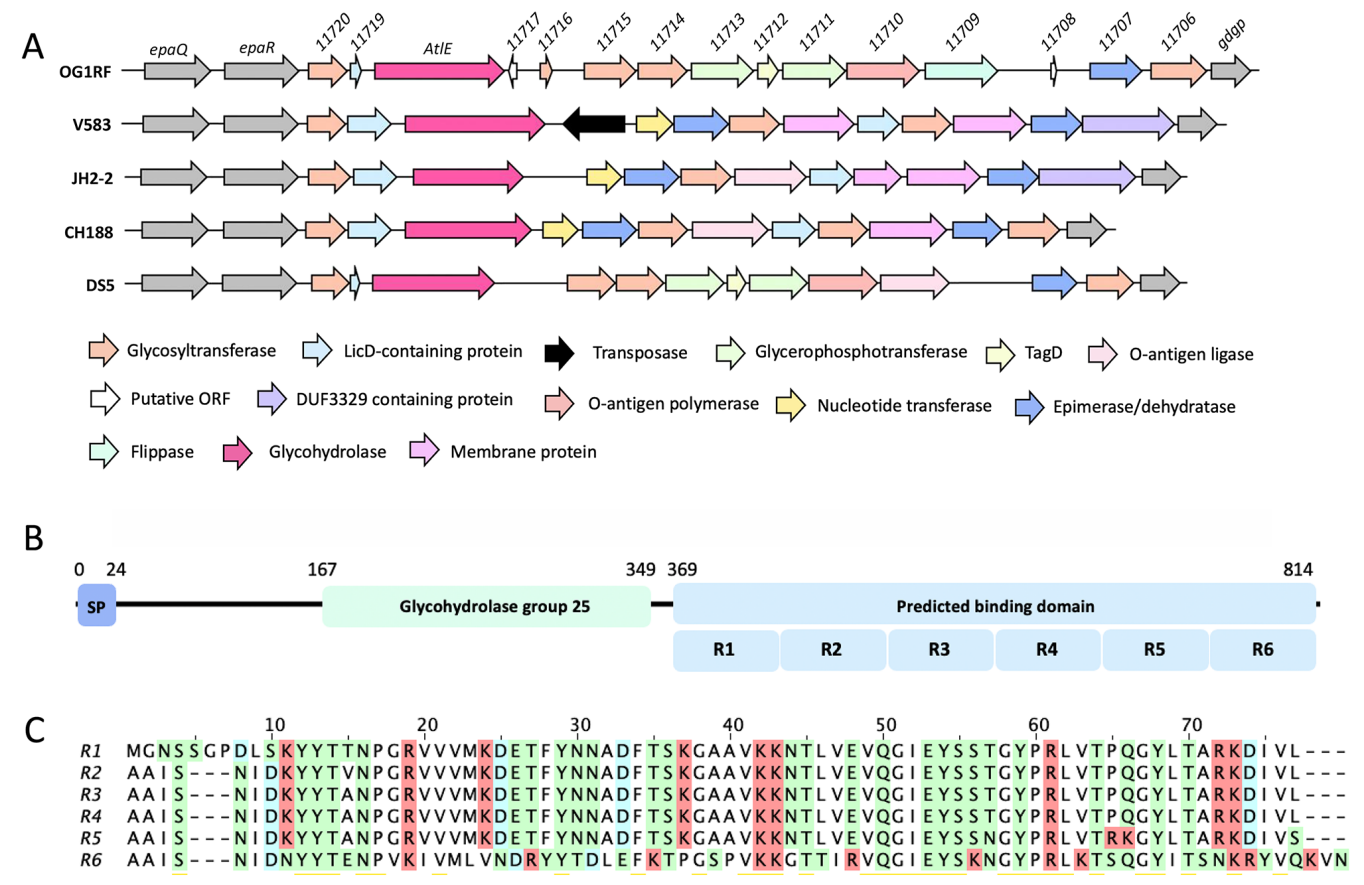
**Schematic representation of the variable locus which encodes AtlE, and domain organization of the enzyme.** (A) *atlE* is present in all *E. faecalis* strains within the EPA variable genetic locus, flanked by two conserved genes encoding a glycosyltransferase (*epaR*) and a glycerophosphodiester phosphodiesterase *(gdgp)*. Five model *E. faecalis* strains are shown as an example (La Rosa et al. 2015). Genes are coloured accorded to predicted function. (B) AtlE in OG1RF is predicted to contain a signal peptide (SP; residues 1–24), a glycohydrolase group 25 (GH25) domain (residues 167–349), and a predicted binding domain (residues 369–814) consisting of six repeating units (R1-R6). (C) The R1-R6 domains are highly conserved. Positively charged residues are highlighted in red, negative residues are highlighted in blue, and polar residues are highlighted in green

*E. faecalis* is a leading cause of nosocomial infection, causing life-threatening infections in immunocompromised patients and patients with antibiotic-induced dysbiosis (Arias and Murray 2012; Diekema et al. 2019). The virulence of *E. faecalis* and its resistance to antimicrobials is associated with the presence of a surface rhamnopolysaccharide called the Enterococcal Polysaccharide Antigen (EPA; Xu et al. 2000). EPA is made of a rhamnan backbone substituted by “decorations” that underpin its biological activity (Smith et al. 2019). Although EPA decorations vary between strains, one gene encoding a putative peptidoglycan hydrolase (AtlE) is ubiquitous, suggesting an important contribution to EPA activity (Fig. 1 A). AtlE contains a signal peptide, a glycosyl hydrolase family 25 (GH25) catalytic domain, and a C-terminal domain with six conserved repeats (R1-R6) of unknown function (Fig. 1B). The major peptidoglycan hydrolase expressed in *E. faecalis*, AtlA (Qin et al. 1998; Mesnage et al. 2008), also has six C-terminal repeats that are essential for substrate binding and overall catalytic activity (Eckert et al. 2006). The R1-R6 domains of AtlE are therefore likely to also be involved in substrate binding and may ultimately contribute towards the biological activity of AtlE.

Here, we report the complete assignment of one of the conserved repeats from the C-terminal domain of OG1RF AtlE, and the corresponding structure prediction. These NMR assignments represent a step forward towards the identification of the cell wall motif recognised by the R1-R6 domain in AtlE, and of how this domain contributes to *E. faecalis* virulence.

## Methods and experiments

### Protein expression and purification

Due to the high sequence similarity of the OG1RF R1-R5 sequences (Fig. 1 C), the most divergent repeat (R6, residues 742–819 of AtlE) was selected for cloning, expression, and purification. The sequence encoding the R6 domain was PCR amplified using oligonucleotides R6_Fw (ATACCATGGCAGCAATCAGTAATATTGACAACTA) and R6_Rev (ATGGGATCCATTCACTTTTTGTACATAACGTTTATTTG) and cloned into pET2818 using NcoI and BamH1 sites to generate pET2818_R6. The plasmid encodes an 86-residue polypeptide with a hexahistidine tag at the C-terminus.

*E. coli* Lemo21(DE3) cells were transformed with the pET2818_R6 construct for protein overexpression and purification. Cells were cultivated at 37 °C, shaking, in M9 media, containing 2 g/L of ^13^C-glucose and 1 g/L of ^15^NH_4_Cl as the only carbon and nitrogen sources, respectively. When an OD_600_ of 0.7 was reached, R6 expression was induced by the addition of 1 mM isopropyl β-D-1-thiogalactopyranoside at an incubation temperature of 25 °C. After 12 h, cells were harvested by centrifugation at 6000 ⋅ g for 15 min at 4 °C. Pellets were resuspended in 20 mL of buffer A (50 mM phosphate, 300 mM NaCl, pH 7.5) supplemented with a Roche cOmplete™ EDTA free protease inhibitor tablet. Cells were lysed by sonication and spun at 30,000 ⋅ *g* for 30 min at 4 °C. The supernatant was then loaded onto a 5 mL HisTrap affinity column and equilibrated in five column volumes of buffer A. The His-tagged R6 was eluted using a 150 mL 0–100% gradient of buffer A containing 500 mM imidazole and concentrated using a Vivaspin 10,000 MWCO centrifugal concentrator (Generon, Slough, UK). Gel filtration was performed on the concentrated protein using a Superdex 75 26/200 column pre-equilibrated in buffer C (40 mM phosphate buffer, pH 6.0). Fractions containing R6 were collected and concentrated as described above to a final concentration of 1.2 mM.

## NMR experiments

All NMR experiments were recorded at 298 K using a Bruker Neo 600 MHz NMR spectrometer with a 5 mm TCI cryoprobe running TopSpin version 4.0.5. NMR experiments were performed in 5-mm NMR tubes containing 1 mM R6, 1 mM trimethylsilylpropanoic acid (TSP), 2 mM sodium azide, and 10% v/v ^2^H_2_O, in 40 mM phosphate buffer at pH 6.0, with a total volume of 550 µL. Two-dimensional ^15^N-^1^H and ^13^C-^1^H HSQC, and an assortment of three-dimensional NMR experiments, HNCO, HNCACO, HNCA, HNCOCA, HNCACB, HNCOCACB, HCCH-TOCSY and CCH-TOCSY, were performed for the assignment of R6. The assignment of arginine N^ε^-H side-chain resonances required an additional three-dimensional TOCSY-HSQC experiment, with a mixing time of 120 ms. Using information obtained from the assigned ^15^N-^1^H HSQC spectrum, HA and HB resonances were assigned from a three-dimensional HBHA(CO)NH experiment, which were then used with the HCCH-TOCSY and CCH-TOCSY for sidechain assignments. In all cases, standard Bruker pulse sequences were used. The ^1^H chemical shifts were referenced according to the internal ^1^H signal of TSP resonating at 0.00 ppm. ^13^C and ^15^N chemical shifts were then referenced indirectly according to nuclei-specific gyromagnetic ratios.

## Extent of assignment and data deposition

Chemical shifts corresponding to the ^1^H_N_, ^15^N, ^13^C^α^, ^13^C^ß^,^13^C^’^ of the R6 backbone were assigned using the standard triple resonance approach (Gardner and Kay 1998). Spectra were processed and analysed using TopSpin version 4.0.2 and FELIX (FELIX NMR, Inc.). The “asstools” assignment program (Reed et al. 2003) was employed to align and match spin systems to the R6 sequence for the assignment of the R6 backbone. ^1^H^α^, ^1^H^ß^ and arginine side chain resonances (N^ε^-H^ε^) were assigned manually following the method of Ohlenschläger et al. (1996), using the R6 backbone assignments as reference. Sidechain resonances were assigned using HCCH-TOCSY and CCH-TOCSY experiments.

**Fig. 2:**
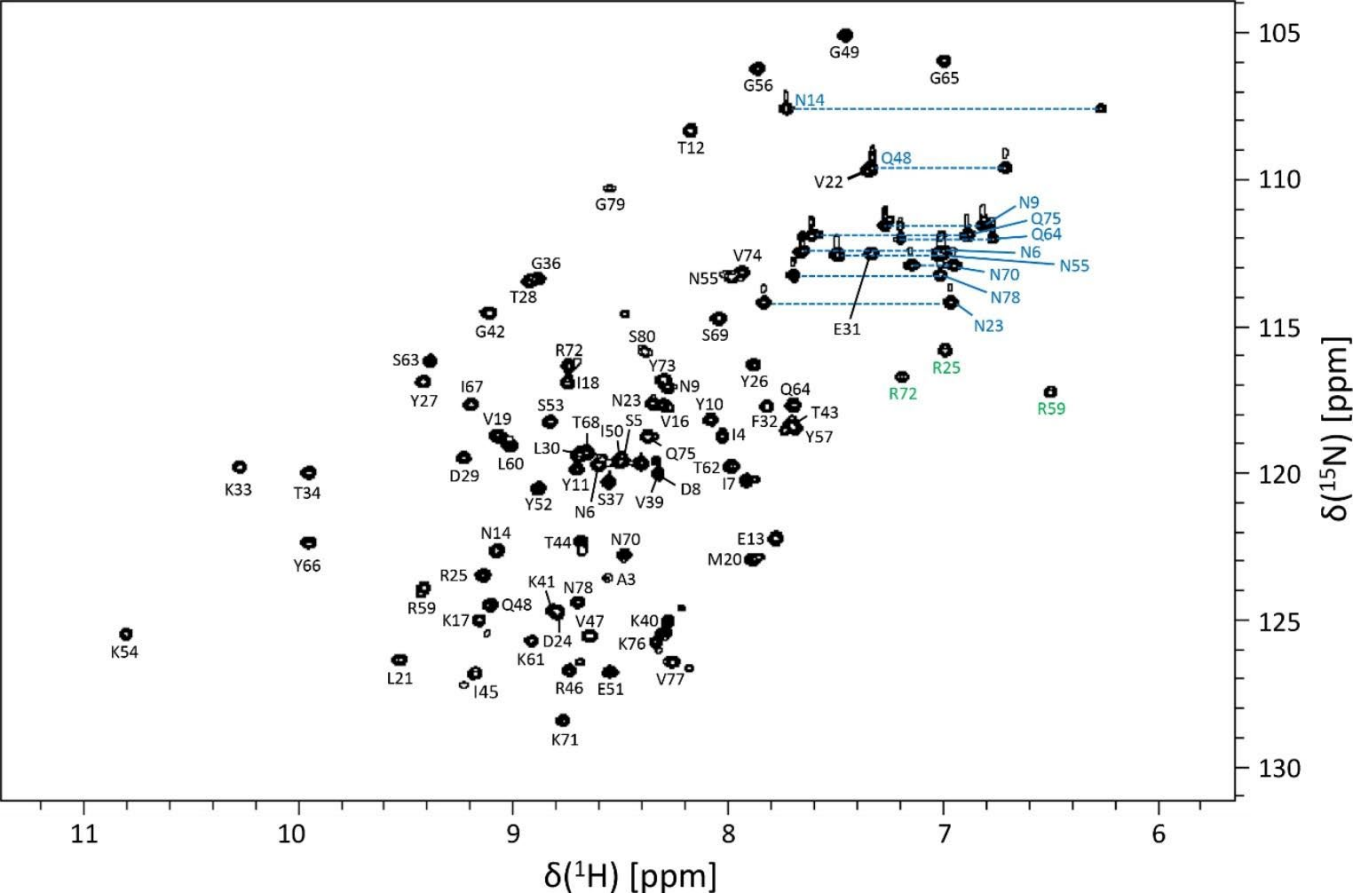
Assigned ^15^N-^1^H HSQC spectrum of the AtlE R6 sequence, at 298 K in 40 mM phosphate buffer (pH 6), 90% H_2_O, 10% D_2_O. Signals are labelled according to amino acid one letter code and position in the primary sequence. Backbone NH signals are in black, asparagine and glutamine side chain NH_2_ pairs are in blue, connected by a dashed line, and arginine NHε signals are in green. The NHε signal of R46 is not shown, due to low signal on this plot

Figure 2 shows the assigned ^15^N HSQC spectrum for the recombinant R6 protein. The spectrum is of high resolution with clear, well-defined peaks. Excluding the “difficult” signals (N-terminal residue and His-tag, non-protonated aliphatic and aromatic C and N, Argη, Lysζ), 98.7% of all backbone ^15^N and amide protons were assigned (missing only A2), 100% of all C^α^, C^β^, C^’^, H^α^ and H^β^ backbone signals were obtained, as well as 100% of all asparagine sidechain N^δ^, H^δ1^ and H^δ2^ signals, 100% of all glutamine N^ε^, H^ε1^ and H^ε2^, and 100% of arginine N^ε^-H^ε^ signals. 89.5% of sidechain signals were assigned, with most of the missing signals being from aromatic rings. Arginine side chain signals (Fig. 2, green) are folded in the nitrogen dimension. The full list of assigned shifts can be found within the BioMagResBank (http://www.bmrb.wisc.edu) under accession number 51184.

**Fig. 3:**
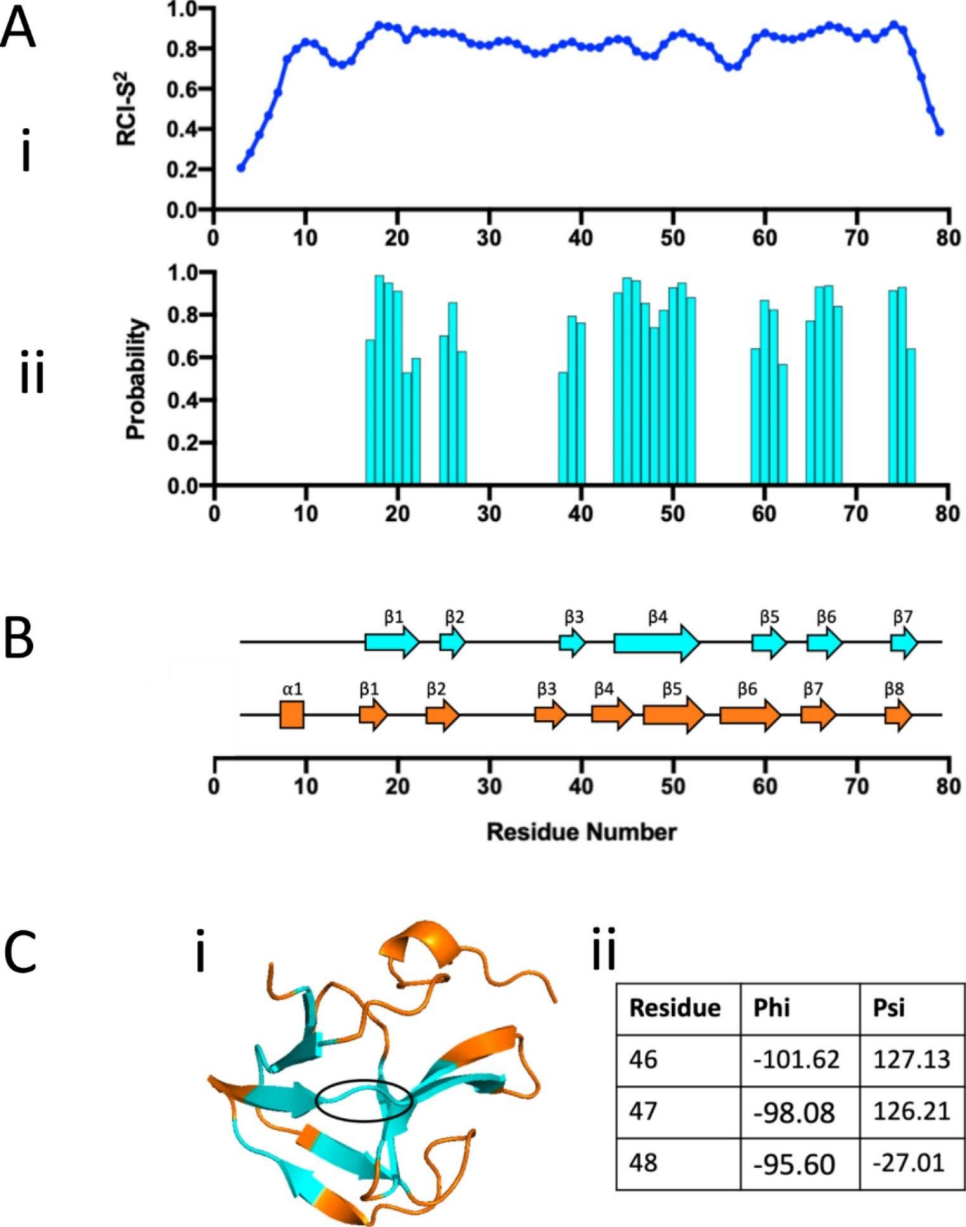
Protein dynamics and structure prediction of the AtlE R6 sequence. TALOS-N and the reported backbone chemical shifts were used to (i) calculate the random coil index order parameter (RCI-S^2^), and (ii) predict the secondary structure of each R6 residue. Using TALOS-N, R6 was predicted to contain only β-sheets. (B) AF2 was also employed to predict the secondary structure of R6 (orange), using the primary sequence alone, and compared against the TALOS-N prediction (cyan). In the case of AF2, one ⍺-helix (rectangle) and seven β-sheets (arrows) were predicted to make up the R6 secondary structure. (C) Some differences were observed between the TALOS-N and AF2 secondary structure predictions. (i) Specifically, the AF2 structure (orange) predicted a break in the β-sheet between residues 46–48 (circled in black), which is not seen in the TALOS-N prediction (cyan). (ii) Torsion angles predicted by TALOS-N at residues 46, 47 and 48

The TALOS-N webserver (Shen and Bax 2013) was then used to predict the dynamics and secondary structure of R6 from the reported backbone chemical shifts (Fig. 3 A). The random coil index values (RCI-S^2^) shown in Fig. 3Ai indicate R6 may have three dynamic regions, specifically at the N-terminus, C-terminus and between residues 54 to 58. Secondary structure prediction (Fig. 3Aii) suggests R6 to contain seven β-sheets (β1: K17-V22, β2: R25-Y27, β3: P38-K40, β4: T44-Y52, β5: R59-T62, β6: G65-T68, β7: V74-K76), with a flexible region predicted to fall between β4 and β5.

Alphafold-2 (AF2) is another protein structure predictor which allows the accurate prediction of protein structures using only the primary sequence (Jumper et al. 2021). In theory this platform could therefore be used in tandem with TALOS-N as a validation method. AF2 predicted a similar overall protein secondary structure for R6, as compared to the TALOS-N output, as shown in Fig. 3B. Whilst eight β-sheets were predicted rather than seven (β1: K17-M20, β2: D24-Y27, β3: G36-V39, β4: G42-R46, β5: Q48-S53, β6: G56-T62, β7: G65-T68, β8: V74-K76), the vast majority were in very similar positions to those predicted by TALOS-N. Of interest AF2 predicts a break in the fourth β-sheet predicted by TALOS-N, between residues 46–48. This discrepancy occurs between two anti-parallel β-sheets in the AF2 tertiary structure (Fig. 3Ci) and may be explained by the dihedral angles predicted by TALOS-N within this region (Fig. 3Cii). Whilst residues 46 and 47 have ϕ and ψ angles characteristic of a β-sheet conformation, residue 48 does not. Taking this into account with the AF2 predicted tertiary structure, this suggests R6 does have a small break within the TALOS-N predicted β4.

In summary, our results suggest that we have produced a reliable prediction of the dynamics and secondary structure of R6, and that AF2 can be used as a tool to complement chemical shift-based protein structure predictions. The assignments and structural details reported here will be used to explore the binding of this domain to the cell wall, to begin to understand the biological activity of AtlE, and ultimately, its potential contribution to *E. faecalis* virulence.

## Data Availability

The NMR chemical shift assignments have been deposited at the Biological Magnetic Resonance Data Bank (http://www.bmrb.wisc.edu) under the BMRB accession number 51184.
